# A content analysis of dissemination and implementation science resource initiatives: what types of resources do they offer to advance the field?

**DOI:** 10.1186/s13012-017-0673-x

**Published:** 2017-11-21

**Authors:** Doyanne Darnell, Caitlin N. Dorsey, Abigail Melvin, Jonathan Chi, Aaron R. Lyon, Cara C. Lewis

**Affiliations:** 1 Department of Psychiatry and Behavioral Sciences, University of Washington, 325 Ninth St. Box 359911, Seattle, WA 98104 USA; 2 Kaiser Permanente Washington Health Research Institute, MacColl Center for HealthCare Innovation, 1730 Minor Ave St. 1600, Seattle, WA 98101 USA; 3 Department of Psychological and Brain Sciences, Indiana University, 1101 E 10th Street, Bloomington, IN 47405-7007 USA

**Keywords:** Dissemination, Implementation science, Translational research, Evidence-based practice, Behavioral health, Mental health, Physical health

## Abstract

**Background:**

The recent growth in organized efforts to advance dissemination and implementation (D & I) science suggests a rapidly expanding community focused on the adoption and sustainment of evidence-based practices (EBPs). Although promising for the D & I of EBPs, the proliferation of initiatives is difficult for any one individual to navigate and summarize. Such proliferation may also result in redundant efforts or missed opportunities for participation and advancement. A review of existing D & I science resource initiatives and their unique merits would be a significant step for the field. The present study aimed to describe the global landscape of these organized efforts to advance D & I science.

**Methods:**

We conducted a content analysis between October 2015 and March 2016 to examine resources and characteristics of D & I science resource initiatives using public, web-based information. Included resource initiatives must have engaged in multiple efforts to advance D & I science beyond conferences, offered D & I science resources, and provided content in English. The sampling method included an Internet search using D & I terms and inquiry among internationally representative D & I science experts. Using a coding scheme based on a priori and grounded approaches, two authors consensus coded website information including interactive and non-interactive resources and information regarding accessibility (membership, cost, competitive application, and location).

**Results:**

The vast majority (83%) of resource initiatives offered at least one of seven interactive resources (consultation/technical assistance, mentorship, workshops, workgroups, networking, conferences, and social media) and one of six non-interactive resources (resource library, news and updates from the field, archived talks or slides, links pages, grant writing resources, and funding opportunities). Non-interactive resources were most common, with some appearing frequently across resource initiatives (e.g., news and updates from the field).

**Conclusion:**

Findings generated by this study offer insight into what types of D & I science resources exist and what new resources may have the greatest potential to make a unique and needed contribution to the field. Additional interactive resources may benefit the field, particularly mentorship opportunities and resources that can be accessed virtually. Moving forward, it may be useful to consider strategic attention to the core tenets of D & I science put forth by Glasgow and colleagues to most efficiently and effectively advance the field.

## Background

In the last decade, numerous efforts to advance the science of dissemination and implementation (D & I) have emerged globally. Conferences to disseminate scientific innovations occur with regularity around the world, D & I science training is available through specialized institutes, and D & I science is seen as global public health priority [1], receiving support from funding agencies worldwide [2]. The proliferation of such efforts is a testament to the widespread recognition that knowledge of what is effective for patients is frequently not translated into real world practice [3–5] and that there appears to be a lack of empirical scrutiny applied to methods for disseminating and implementing evidence-based practices (EBPs) [6–8].

Anecdotally, we have observed a similar proliferation of organized efforts to disseminate knowledge and skills for D & I science, which we refer to as “D & I science resource initiatives.” The motives for developing D & I science-focused resource initiatives likely vary greatly, but may include reasons such as expanding professional networks to identify collaborators or to facilitate institutional support and interest in D & I (e.g., the US Clinical and Translational Science Awards; [9]). These resource initiatives provide useful resources to the D & I science community, which ultimately bodes well for improvements in human health. However, the impact of these resource initiatives is dependent on how well they can reach their target audiences and whether potential participants fully understand their relative merits. Further, the diffusion of efforts to advance D & I science without attention to how these resources complement each other could unwittingly stymie scientific progress [10]. For example, concerns about inefficient use of resources prompted researchers at National institutes of health (NIH) to offer guidance to D & I scientists on how future efforts can harness resources to most effectively advance the field [10]. For example, one suggested means of improving relevance and rigor of research is for scientists to engage in collaborations with community stakeholders. Such guidance may apply equally well to how D & I science resource initiatives approach the selection of their activities and resources offered.

It is not clear how D & I science resource initiatives prioritize activities to pursue and resources to offer to the public or members, and we are not aware of any effort to summarize the activities and the resources they offer to the field. Therefore, the main objectives of the present study were to survey the global landscape of current D & I science resource initiatives, identify types of resources they offer, and characterize their accessibility to the D & I science community. Given the value of practice and policy collaborators to support contextually relevant research questions and methods [7], we aimed to identify whether resource initiatives incorporated non-researcher stakeholders and whether resources varied between these types of resource initiatives. Additionally, we aimed to specify whether resource initiatives focused on D & I as they pertain to advancing EBPs for physical versus behavioral health (i.e., both mental and substance use) problems. This is a meaningful distinction given the preponderance of EBPs for behavioral health is complex psychosocial interventions that require unique ways of measuring fidelity and the involvement of purveyors, for example, that often does not occur in other disciplines [3]. We then explored whether behavioral health versus health focused resource initiatives varied in the types of resources they offer.

## Methods

### Design

We conducted a web-based and expert-informed search and content analysis to examine characteristics and available resources of D & I science resource initiatives using public website information and both a priori and an emergent, grounded approach [11]. Data represents publicly available website information accessed between October 2015 and March 2016.

#### Sampling strategies

To obtain the sample of resource initiatives, we utilized two search methods: (1) searching the Internet for websites and (2) inquiring about known resource initiatives from internationally representative D & I science experts and their colleagues (i.e., snowball sampling). For the first method, we used Google Advanced Search to find publicly available websites. Two members of the research team independently inputted search strings comprised of common D & I science-related terms using phrases taken from Proctor and colleagues’ review of D & I concepts (see Table 1) [12]. Search string examples include “implementation science” and “knowledge translation,” the latter of which highlights our efforts to include international resource initiatives given that is the term more frequently used in Canada [13]. These terms can be found in Table 1. This search process continued until both members perceived saturation (i.e., search terms no longer produced novel resource initiatives). This search method resulted in a total of 97 resource initiatives for potential study inclusion.

**Table 1:** Examples of search terms used to identify study resource initiatives via Google

	Search term string
1.	Behavioral health research
2.	Behavioral health treatment research
3.	Conference for implementation dissemination
4.	Conference for implementation dissemination research
5.	Conference for implementation dissemination science
6.	Dissemination implementation behavioral health practice research
7.	Dissemination implementation research behavioral health
8.	Dissemination implementation research evidence based practices
9.	Dissemination implementation research resource initiative
10.	Dissemination implementation science
11.	Dissemination implementation science conference
12.	Evidence based behavioral health practice research
13.	Implementation dissemination EBPS research
14.	Implementation dissemination resource initiative
15.	Implementation dissemination research group/agencies
16.	Implementation research
17.	Implementation research conference
18.	Implementation research in mental health services
19.	Implementation science research methods
20.	Knowledge translation resource initiative
21.	Knowledge translation research conference
22.	Mental health implementation science
23.	Mental health knowledge translation resource initiative
24.	Mental health services research
25.	Mental health treatment effectiveness research
26.	Mental health treatment implementation dissemination
27.	Mental health treatment research conference

For the second method, we employed a snowball-sampling approach. Specifically, emails detailing the purpose of the study, as well as our resource initiative inclusion criteria (see below), were sent out to over 200 members of the Society for Implementation Research Collaboration’s (SIRC) Network of Expertise (NoE; [14]). SIRC’s NoE is comprised of implementation science researchers at various career stages, including established implementation scientists, new investigators, and student members. To supplement the NoE emails, the same content was posted publicly on SIRC’s website. Finally, this same request for resource initiatives was sent to an international advisory board of nine implementation scientists representing the following countries: Australia, Canada, England, Germany, and Sweden. This second search method resulted in a total of 33 additional resource initiatives for potential study inclusion.

#### Inclusion criteria

We defined resource initiatives as organized efforts to disseminate knowledge and foster skills for advancing D & I science (not translational science broadly) via provision of resources to the research community broadly (e.g., excluding academic centers that only provide resources to the local institution). We were particularly interested in efforts that engaged in multiple strategies for advancing D & I science and therefore excluded efforts that reflect *only* a conference or conference series, a training institute, or funding mechanism. Resource initiatives could advance D & I science through conducting their own research, promoting D & I science, or promoting use of science-informed D & I practice. Additionally, the content website had to be available in English.

All initial 130 potential resource initiatives were rated for inclusion by two independent raters; any discrepancies were resolved by the research team. The final sample included 42 resource initiatives that met inclusion criteria. The names of resource initiatives, their corresponding website, and countries of origin are noted in Table 2. The majority of exclusions were due to not advancing D & I science beyond the organization of a conference or beyond the scope of a single institution.

**Table 2:** Study dissemination and implementation science resource initiatives, website, and countries of origin, *N* = 42

Initiative	Origin	Consultation or Technical Assistance	Mentorship	Workshops	Workgroups	Networking	Conference	Social media	D & I resource library	News and updates from the field	D & I archived talks/slides	D & I link page on website	Grant writing resources	Funding opportunities	Stakeholder involvement	Physical health focus	Membership
Academy for Health Improvement (AHI) https://www.a4hi.org/home/index.cfm	USA				X		X	X	X	X	X	X				X	X
Academy Health http://www.academyhealth.org/	USA			X	X		X	X	X	X	X	X		X	X	X	X
Active Living Research http://activelivingresearch.org/	USA	X					X	X	X	X	X		X	X			
Advancing Integrated Mental Health Solutions (AIMS) Center https://aims.uw.edu	USA	X		X					X	X		X			X		
Alliance for Health Policy and Systems Research (AHPSR) http://www.who.int/alliance-hpsr/en/	Switzerland								X	X	X		X	X	X	X	
Australian Research Alliance for Children & Youth (ARACY) http://www.aracy.org.au/	Australia			X	X			X	X	X	X	X			X		X
Behavioral Health Research Center of the Southwest (BHRCS) http://www.bhrcs.org/	USA	X										X			X		
Blueprints for Healthy Youth Development http://www.colorado.edu/cspv/blueprints/	USA						X				X	X		X	X		
Cancer Implementation Science Community of Practice http://www.cancerimplementationscience.org.au/	Australia		X	X	X			X		X	X	X		X		X	X
Center for Community Health and Development and Community Toolbox http://ctb.ku.edu/en	USA			X	X			X	X	X	X	X	X		X	X	
Center for Health Innovation & Implementation Science http://www.hii.iu.edu/	USA	X		X		X				X						X	
Center for Implementation-Dissemination of Evidence-Based Practices Among States http://www.ideas4kidsmentalhealth.org	USA	X	X	X	X					X	X	X			X		X
Center for Quality Assessment and Improvement in Mental Health http://www.cqaimh.org/index.html	USA								X	X	X	X			X		
Centre for Evidence-Based Medicine http://www.cebm.net/	England			X		X	X	X	X	X	X	X		X	X	X	
Consolidated Framework for Implementation Research http://www.cfirguide.org/index.html	USA								X			X				X	
European Implementation Collaborative http://www.implementation.eu/	Europe				X			X	X	X		X			X		X
Global Implementation Society http://gis.globalimplementation.org/	Not specified															X	X
Guidelines International Network http://www.g-i-n.net/home	Scotland			X	X	X	X	X	X	X	X	X			X	X	X
Improvement Science Research Network (ISRN) http://isrn.net/	USA			X	X		X	X	X	X	X	X	X		X	X	X
International Society for Evidence-Based Health Care (ISEHC) http://www.isehc.net/	Multinational						X	X	X	X		X				X	X
Knowledge Translation Canada http://ktclearinghouse.ca/ktcanada	Canada	X	X		X		X	X		X	X	X		X	X	X	X
Knowledge Translation for Disability and Rehabilitation Research (KTDRR) http://ktdrr.org/index.html	USA	X		X	X		X	X	X	X	X	X			X		
Knowledge Utilization Studies Program (KUSP) http://www.kusp.ualberta.ca/	Canada						X		X	X	X	X			X	X	
Mental Health Commission of Canada—Knowledge Exchange Centre http://www.mentalhealthcommission.ca/English/focus-areas/knowledge-exchange-centre	Canada			X	X	X			X		X	X			X		X
National Cancer Institute—Division of Cancer Control and Population Sciences http://cancercontrol.cancer.gov/is/	USA									X	X	X	X	X		X	
National Implementation Research Network (NIRN) http://nirn.fpg.unc.edu/	USA							X	X	X	X	X			X	X	
National Institute for Health Research (NIHR) http://www.nihr.ac.uk/	UK			X		X		X	X	X		X	X	X	X	X	X
North Carolina Translational & Clinical Sciences Institute Implementation Science Exchange http://impsci.tracs.unc.edu/	USA					X		X			X	X	X				
Primary Health Care Research & Information Science (PHCRIS) http://www.phcris.org.au/aboutus/	Australia					X	X	X	X	X	X	X	X		X	X	
Reach Effectiveness Adoption Implementation Maintenance (RE-AIM) http://re-aim.org/	USA									X	X	X				X	
Society for Implementation Research Collaboration https://societyforimplementationresearchcollaboration.org/	USA		X	X			X	X	X	X		X	X		X		X
Society for Implementation Science in Nutrition (SISN) http://www.implementnutrition.org/	Italy														X	X	
Society for Medical Decision Making (SMDM) http://smdm.org/	USA	X	X	X	X	X	X	X		X	X	X	X		X	X	X
Society for Prevention Research (SPR) www.preventionresearch.org	USA			X	X	X	X	X		X		X			X		X
The Health Foundation http://www.health.org.uk/	UK				X	X	X	X	X	X	X		X	X	X	X	
Translating Research Into Action (TRAction) Project http://www.tractionproject.org/	USA							X	X	X	X	X	X	X	X	X	
Triangle Global Health Consortium http://www.triangleglobalhealth.org	USA		X	X	X	X	X	X		X	X	X			X	X	X
UCLA Clinical and Translational Science Institute http://www.ctsi.ucla.edu/patients-community/pages/dissemination_implementation_improvement	USA						X				X	X	X			X	X
UK Implementation Network http://uk-in.org.uk/index.html	UK			X	X			X		X					X		X
University of Connecticut Institute for Collaboration on Health, Intervention, and Policy http://chip.uconn.edu/	USA			X	X			X	X	X	X	X	X	X	X	X	X
US Department of Veterans Affairs—Quality Enhancement Research Initiative (VA QUERI) http://www.queri.research.va.gov/	USA							X	X	X	X	X	X	X	X	X	
UW Institute for Clinical and Translational Research (UW ICTR) https://ictr.wisc.edu/Home	USA	X				X		X		X	X	X	X	X	X	X	X

X = Present

### Units of analysis

Units of analysis for this study included D & I science resource initiative websites, including information from the main website and any related conference websites if applicable. Each page that contained the same base URL was eligible for coding purposes (described below under the “Coding Process” section).

### Coding scheme

We developed codes for resource initiative characteristics based on a priori theoretical interests (see Table 3 for code definitions). Codes for resources were also identified by an emergent process, whereby we observed types of resources offered by resource initiatives during initial review of the websites. Resources that warranted coding emerged based on an anticipated interest of the resource to the field of D & I science or commonality among the resource initiatives. Additionally, we qualified the degree to which resources may be more or less available to the larger D & I science community through the use of sub-codes. Where relevant, we gathered qualitative data to describe the nature of coded resources.

**Table 3:** Definitions of content analysis codes: interactive resources, non-interactive resources, and resource initiative characteristics

Code	Definition
Interactive resources	
Consultation or technical assistance	Technical assistance (TA) is the process of providing targeted support to an organization with a development need or problem. TA may be delivered in many different ways, such as one-on-one consultation, small group facilitation, or through a web-based clearinghouse. The help given should enhance the users’ knowledge or ability to carry out dissemination & implementation (D & I) science or practice. It is in depth and focused on a particular issue unlike mentorship, which focuses on a person.
Mentorship	The resource initiative organizes an ongoing relationship for the individual seeking support and a content expert to realize individualized goals.
Workshops	Workshops are typically one-time trainings in a particular content or a resource offered by a resource initiative either in-person or virtually. The workshop must involve interaction between the participants and/or the instructors.
Workgroups	The resource initiative organizes a group of individuals who share interest in a particular content, mission, or resource development process (typically they set workgroup goals) to facilitate live interaction and development of a project.
Networking	The resource initiative creates a virtual or physical time and space for individuals with shared interests to discuss their synergy.
Conference	The organization arranges a large-scale meeting on a regularly-occurring basis to bring together members, professionals in the field, stakeholders, and other interested individuals to present new information and study findings with the goal of advancing the field.
Social media	The resource initiative has links to its self-maintained social media profiles (e.g., Facebook, Twitter, LinkedIn).
Non-interactive resources	
D & I resource library	The resource initiative has gathered at least 10 established resources (e.g., instrument/measures, white papers, fact sheets, journal articles, tools, information about methods) that researchers and stakeholders may access for information and/or use. List of resources must be more though or comprehensive than simply a link.
News and updates from the field	The resource initiative purposefully reports on or distributes current, updated, and relevant information from the field such as publications, job ads, research findings, etc. in the form of a listserv, newsletter, blog, and/or hard copy mailings.
D & I archived talks/slides	The resource initiative provides access to D & I videos, talks, slides. This includes webinars.
D & I link page on website	The resource initiative offers a link page with information related to external resources and opportunities, notably substantive summaries of training opportunities, exhaustive listing of conferences, etc.
Grant writing resources	The resource initiative offers resources related to writing grants, with the goal of providing guidance (e.g., annotated program announcements, offering examples of funded grants, highlighting funding agencies, etc.)
Funding opportunities	Funding opportunities (e.g., scholarships and grants) for individuals or groups are available through the organization, usually in the way of supporting research endeavors.
Resource initiative characteristics	
Stakeholder involvement	Anyone affected by implementation, but not a scientist, informed, shaped, produced, or contributed to the resources.
Healthcare domain	The mission or relevance of activities is focused on D & I for EBPs for physical health or behavioral health.
Membership	This variable indicates whether the resource initiative has a membership option.

#### Main codes

Codes for characteristics of resource initiatives included whether the resource initiative was focused on implementing EBPs targeting physical health, behavioral health (i.e., mental health and/or substance use disorder) EBPs, or a combination of both. Additionally, we captured whether the resource initiative included participation of a non-researcher stakeholder and whether there was an option to become a formal member of the resource initiative (either for a fee or free).

The types of resources provided by resource initiatives were classified into interactive (i.e., require participation of two parties in some way) and non-interactive (i.e., accessible by one party). Interactive resources included (1) consultation/technical assistance, (2) mentorship, (3) workshops, (4) workgroups, (5) networking, (6) conferences, and (7) social media. Non-interactive resources included (1) a resource library, (2) news and updates from the field, (3) archived talks or slides (includes webinars), (4) links pages, (5) grant writing resources, and (6) funding opportunities.

#### Sub-codes

As applicable, sub-codes identified whether a resource was accessible to only members or the general public, associated with a fee, required a competitive application, or was available in-person, virtually, or both.

### Coding process

#### Coder training and pilot coding

The research team completed three rounds of pilot coding, which resulted in iterative modifications to the coding scheme to enhance reliability of its application and inductively inform the coding scheme. Two coders were trained in the final coding scheme as well as how to navigate the resource initiative’s website to obtain information relevant to the coding scheme. This included instruction to (1) open all of the links on a page as soon as a new page is opened, (2) go through each link on a page before progressing to the next page, and (3) work from top-to-bottom and left-to-right when opening links and reading through a webpage. Coders only included information present on the resource initiative’s main URL. The only exception to this was if the resource initiative’s conference had a unique URL, in which case the information from the conference website was also included. Two coders independently coded each of the 42 resource initiatives and then met to review and determine final codes based on consensus when coding was discrepant. Each coder coded the website within 3 months of the other coder.

#### Inter-rater reliability

We estimated inter-rater reliability for each main code (i.e., resources and characteristics). Because sub-codes were dependent on the main codes, and some main codes occurred infrequently, we did not calculate estimates of inter-rater reliability for sub-codes. After independent coding had been completed for approximately half of the resource initiatives, the rates of agreement calculated for each main code were reasonable, ranging from 67 to 90%.

### Plan of analysis

We observed frequencies of resource initiative characteristics and resources, as well as the frequencies of sub-codes, when applicable. We explored bivariate relations between resource initiative characteristics and available resources, as well as the relations between resource initiative characteristics, using Chi-square tests of independence, with *p* < .05 indicating statistical significance. As these analyses were exploratory in nature, we did not use a corrected significance level for the large number of statistical tests.

## Results

### Resource initiative characteristics

Table 4 presents the results of the coding process described above. Per websites, the majority of resource initiatives *(n* = 31, 74%) incorporated non-researcher stakeholder involvement, and a little more than half focused solely on D & I science as it relates to physical health EBPs (*n* = 25, 60%). Half of the resource initiatives offered formal membership (*n* = 21, 50%), and when offered, membership was associated with a fee half of the time (*n* = 10, 24% of all resource initiatives). We did not observe bivariate relationships between the resource initiative characteristics.

**Table 4:** Content analysis coding scheme and frequencies of codes for dissemination and implementation science resource initiatives, *N* = 42

Activity/feature	Total (% of *N*)	Cost *n* (% of total)				Accessibility—membership^a^ *n* (% of total)				Accessibility—competitive application *n* (% of total)				Accessibility—Location *n* (% of Total)			
Interactive resources		P	NP	Both	NS	P	NP	Both	NS	P	NP	Both	NS	V	Ph	Both	NS
Consultation/technical assistance	9 (21)	1 (2)	6 (14)	---	2 (5)	1 (2)	7 (17)	---	1 (2)	1 (2)	7 (17)	---	1 (2)	3 (7)	---	3 (7)	3 (7)
Mentorship	6 (14)	---	4 (10)	---	2 (5)	1 (2)	5 (12)	---	---	1 (2)	4 (10)	---	1 (2)	1 (2)	4 (10)	1 (2)	---
Workshops	19 (45)	10 (24)	6 (14)	2 (5)	1 (2)	---	16 (38)	3 (7)	---	2 (5)	16 (38)	1 (2)	---	3 (7)	11 (26)	5 (12)	---
Workgroups	18 (43)	---	17 (41)	---	1 (2)	7 (17)	9 (21)	1 (2)	1 (2)	---	17 (41)	---	1 (2)	7 (16)	---	7 (17)	4 (10)
Networking	12 (29)	---	12 (29)	---	---	2 (5)	9 (21)	1 (2)	---	---	11 (26)	1 (2)	---	1 (2)	7 (17)	3 (7)	1 (2)
Conference	18 (43)	12 (27)	2 (5)	---	4 (10)	1 (2)	15 (36)	---	2 (5)	1 (2)	16 (38)	---	1 (2)	N/A			
Social media	27 (64)	N/A				N/A				N/A				N/A			
Non-interactive resources		P	NP	Both	NS	P	NP	Both	NS	P	NP	Both	NS	V	Ph	Both	NS
Resource library	25 (60)	---	25 (60)	---	---	1 (2)	23 (55)	1 (2)	---	---	25 (60)	---	---	N/A			
News and updates from the field	34 (81)	---	33 (79)	1 (2)	---	1 (2)	30 (71)	3 (7)	---	---	34 (81)	---	---	33 (79)	---	1 (2)	---
Archived talks/slides	30 (71)	---	29 (69)	1 (2)	---	---	29 (69)	1 (2)	---	---	30 (71)	---	---	N/A			
Link page on website	35 (83)	---	35 (83)	---	---	---	34 (81)	1 (2)	---	---	35 (83)	---	---	N/A			
Grant writing resources	16 (38)	---	16 (38)	---	---	2 (5)	14 (33)	---	---	---	15 (36)	1 (2)	---	N/A			
Funding opportunities	14 (33)	N/A				2 (5)	11 (26)	1 (2)	---	N/A				N/A			

Note: *P* present, *NP* not present, *NS* not specified, *V* virtual, *Ph* physical, *N/A* not applicable/not coded. --- = 0

^a^Present indicates membership required to access the resource, not present indicates the resource is open to the public

### Resources

The vast majority of resource initiatives offered both interactive and non-interactive resources (*n* = 35, 83%). Of the seven types of interactive resources, resource initiatives had an average of 2.57 (SD = 1.81). The most commonly occurring was social media (*n* = 27, 64%). Qualitative data collected on the types of social media indicated that resource initiatives frequently utilized more than one type, with Twitter being the most frequently utilized, (*n* = 25, 60%), followed by Facebook (*n* = 17, 40%) and LinkedIn (*n* = 9, 21%). The presence of workshops (*n* = 19) and conferences (*n* = 18) were also common. Workshops often had a cost associated (*n* = 10) and the majority (*n* = 16) were open to the public. Conferences predominately occurred annually (*n* = 13) were open to the public (*n* = 15) and did not require a competitive application to attend (*n* = 16). The majority of the conferences did have an associated cost (*n* = 12). Workgroups (*n* = 18, 43%) predominately did not have a cost associated with them (*n* = 17). Roughly half of the workgroups did not require resource initiative membership (*n* = 9). The least commonly occurring interactive resources were mentorship (*n =* 6, 14%), followed by technical assistance (*n* = 9, 21%) and networking (*n* = 12, 29%). The locations of interactive resources varied: those most commonly only available in-person were mentorship, workshops, and networking.

Of the six types of non-interactive resources, resource initiatives had an average of 3.67 (SD = 1.52). The most common non-interactive resource was the presence of a links page (*n* = 35, 83%), followed by archived talks and slides (which includes webinars, *n* = 30, 71%) and the organization of news and updates from the field (*n* = 34, 81%). These resources rarely cost anything to the user, required membership, or required a competitive application to access. The less frequent non-interactive resources were grant writing resources (*n* = 16, 38%) and funding opportunities (*n* = 14, 33%). It was rare for grant writing resources to be associated with a cost, be limited to members, or contingent on a competitive application. For most resource initiatives that offered funding opportunities, they made these available to the public (*n* = 11). Additionally, 25 (60%) resource initiatives were found to include a resource library, none of which had an associated cost or application and were largely (*n* = 23) open to the public. Common types of resources included in the library were tools/toolkits, guides/guidelines, articles, reports, and systematic reviews.

### Bivariate relations between characteristics and resources

Resource initiatives with a formal membership option were more likely to have mentorship, workshops, and workgroups and to use social media (see Table 5). There were no differences in resources available based on whether resource initiatives had non-researcher stakeholders involved or whether they had a physical health only focus versus a behavioral health only or combined physical health focus.

**Table 5:** Descriptive statistics for interactive and non-interactive resources by resource initiative characteristics

		Stakeholder involvement		Membership		Healthcare domain	
	Total resource initiatives (*n* = 42)	Present (*n* = 31)	Not present (*n* = 11)	Present (*n* = 21)	Not present (*n* = 21)	PH only (*n* = 25)	BH or BH/PH (*n* = 17)
	*n* (%)	*n* (%)		*n* (%)		*n* (%)	
Interactive resources	35 (83)	28 (90)	7 (64)	20 (95)	15 (71)	19 (76)	16 (94)
Consultation or technical assistance	9 (21)	7 (23)	2 (18)	4 (19)	5 (24)	3 (12)	6 (35)
Mentorship	6 (14)	5 (16)	1 (9)	6 (29)*	0 (0)*	4 (16)	2 (12)
Workshops	19 (45)	17 (55)	2 (18)	14 (67)*	5 (24)*	10 (40)	9 (53)
Workgroups	18 (43)	16 (52)	2 (18)	15 (71)*	3 (14)*	10 (40)	8 (47)
Networking	12 (29)	10 (32)	2 (18)	7 (33)	5 (24)	8 (32)	4 (24)
Conference	18 (43)	14 (45)	4 (36)	11 (52)	7 (33)	13 (52)	5 (29)
Social media	27 (64)	22 (71)	5 (46)	17 (81)*	10 (48)*	16 (64)	11 (65)
Non-interactive resources	40 (95)	30 (97)	10 (91)	20 (95)	20 (95)	23 (95)	17 (100)
Resource library	25 (60)	21 (68)	4 (36)	11 (52)	14 (67)	15 (60)	10 (59)
News and updates from the field	34 (81)	27 (87)	7 (64)	18 (86)	16 (76)	21 (84)	13 (77)
Archived talks/slides	30 (71)	23 (74)	7 (64)	14 (67)	16 (76)	19 (76)	11 (65)
Link page on website	35 (83)	27 (87)	8 (73)	19 (91)	16 (76)	20 (80)	15 (88)
Grant writing resources	16 (38)	12 (39)	4 (36)	7 (33)	9 (43)	10 (40)	6 (35)
Funding opportunities	14 (33)	11 (36)	3 (27)	6 (29)	8 (38)	9 (36)	5 (29)

All variables dichotomous. Bivariate relationships between resources and characteristics assessed for statistical significance using Chi-square tests of independence

*Note: PH* physical health, *BH* behavioral health

*p < .05

## Discussion

We identified 42 resource initiatives globally that provide resources to the English-speaking D & I science community and imagined that this number will continue to increase along with growing enthusiasm for the field. The 42 resource initiatives identified represent a diverse array of organized efforts to advance D & I science that provide a variety of interactive and non-interactive resources to not only researchers but also non-researcher stakeholders.

### Availability of interactive and non-interactive resources

Resource initiatives commonly provide both interactive (i.e., require participation of two or more parties) and non-interactive (i.e., accessible by one party) resources (*n =* 35, 83%). Overall, however, non-interactive resources (e.g., resource libraries, archives, grant-writing resources, news/updates) were more common. This is relatively unsurprising given that these resources are often less expensive and easier to maintain than more interactive resources, which often carry a substantial time commitment for those involved (e.g., mentorship). Other benefits to non-interactive resources are that they are typically available “on demand,” more easily and flexibly accessed, and therefore are potentially more scalable than interactive resources. As a result, non-interactive resources have the potential for greater reach [15] to those in the field of D & I, particularly with regard to increased knowledge about D & I science and practice [7]. Findings from the field, however, have shown us that less interactive approaches to information provision are unlikely to result in the uptake or application of new skills [16], which may limit the impact [15] of non-interactive resources. This makes the availability of interactive resources imperative. Additionally, efforts to innovate on scalable web-based didactic material to target skill development may be warranted to build capacity. Moreover, it may be that certain content is best suited for non-interactive resources (i.e., research findings), whereas other content (i.e., new methodologies) might demand interactive resources such as a workshop.

Interactive resources (e.g., conferences, workshops, mentorship, etc.), which provide opportunities for networking and collaboration, are essential to the promotion of the multidisciplinary, team approach that is often needed in the field of D & I science [17]. Additionally, interactive resources may be more impactful than non-interactive resources, both in terms of skill acquisition as previously mentioned and with regard to professional development. For instance, mentorship is known to be integral to professional development [18], and a recent evaluation of the Implementation Research Institute, a training program for D & I scientists, indicates that mentorship plays an essential role in academic successes of its fellows [19]. However, mentorship was infrequent among the resources offered by resource initiatives (*n =* 6, 14%). This is likely due to the ongoing time commitment required for mentors and mentees and the fact that, to be most effective, D & I science mentors are likely to be drawn from a pool of established experts that remains relatively small. A recent study of D & I scientists indicates many pursue research collaborators to obtain mentorship, but few pursue collaborators to provide mentorship [20]. To match the growing demand for D & I scientists, it is prudent for resource initiatives to expand mentoring opportunities.

Social media was commonly, but not uniformly, used by resource initiatives. Social media is particularly useful for publicizing a resource initiative, promoting the larger D & I science field, and sharing information with and between members or stakeholders who use social media platforms. There is some indication that social media can spark collaborations among researchers, particularly through sites like *ResearchGate* (https://www.researchgate.net/), developed specifically to connect researchers [21]; however, the incremental value of social media for collaborative purposes remains to be seen. Review of the types of social media resource initiatives commonly used indicates most rely on sites designed for primarily social (i.e., Twitter) versus research networking (i.e. LinkedIn) activities. It may be that integrated use of both types of sites, as well as creative integration with other types of resources, may hold the most promise for the impact of social media on D & I science [22].

Interactive workshops were offered by just under half of the resource initiatives and have the potential to develop knowledge and skills related to D & I science, as well as offer enhanced networking opportunities. Although these were predominantly offered in-person, the use of virtual workshops may greatly enhance the reach of these resources, as they can be as effective and well-received by participants as in-person workshops [23]. Conferences were also offered by a substantial minority of resource initiatives. Conferences offer researchers the quintessential opportunity for networking combined with sharing novel research ideas and disseminating research findings. They are highly resource intensive for the resource initiative as well as for attendees, requiring time, finances, and preparation of research presentations but valued by funding agencies (e.g., [24]) as well as the researchers.

Recently, there have been growing concerns about the potential for a widening divide between D & I science and practice in which findings from D & I science are not routinely applied when integrating new innovations into service systems, and concern that D & I science is likely to produce tools and strategies that may be impractical in many service settings [7, 25]. Such a divide runs the risk of replicating the well-documented gap between research and practice in intervention science, which was one of the primary catalysts for the original emergence of the D & I field [26–28]. To address this divide, interdisciplinary conferences that bring together researchers and non-researchers, such as policy makers and practice-based administrators and clinicians who champion EBPs, may be essential. However, non-researchers may be less able than researchers to attend conferences in-person, as their organizations may not have financial resources available for travel and attendance or the resources to allot staff time away from clinical or administrative duties. Conferences that aim to reduce the D & I science-practice divide may benefit from incorporating virtual participation to accommodate attendees who are valued but unable to otherwise attend in-person and ensuring that continuing education credit offerings are abundant.

### Stakeholder involvement

A sizable majority of resource initiatives incorporated both stakeholders who operate in a research or non-research role (*n =* 31, 74%), which may be essential to addressing the aforementioned D & I science-practice divide. Collaborations between D & I scientists and practitioners (i.e., EBP purveyors, intermediaries, or administrators implementing EBPs in the community), decision-makers, and patient stakeholders are particularly important to advance rigor and relevance of the research [10] and policy to support implementation [29]. However, it remains unclear the extent of non-researcher stakeholder involvement in these resource initiatives and the degree to which interactive resources would include both groups for optimal impact and bidirectional learning.

### Membership

Although a minority of resource initiatives provided opportunities for formal membership (*n =* 21, 50%), those that had this characteristic were more likely to offer interactive resources including mentorship, workshops, workgroups, and social media. However, having a formal membership option was not related to non-interactive resources. It may be that interactive resources are more intensive in nature and are best supported by resource initiatives that have the infrastructure that goes along with being able to offer a formal membership and, potentially, receive financial support through that mechanism. Additionally, membership, even when not associated with a cost, as was the case for half of resource initiatives, may be a means of restricting access to what may be more scarce interactive resources.

### Healthcare domain

The specific healthcare domain on which resource initiatives focused (i.e., having a physical health only focus versus a focus on behavioral health only or in combination with physical health) was not related to the type of resources offered. This result aligns well with the common conceptualization that D & I science transcends any particular domain of healthcare or social service [27]. Indeed, most of the existing theories, frameworks, and models [30]; critical predictors of successful D & I [31]; and strategies to support adoption and sustainment are intended to have applicability across a broad range of EBP and practice domains. Increasingly, these commonalities are giving rise to the use of standard language that is readily interpretable and which serves to promote information sharing and collaboration regardless of the backgrounds of professionals or contexts in which they work. Nevertheless, we anticipate that efforts to integrate different healthcare domains within implementation science may continue to face some barriers due to variable terminology, diverse publication outlets, distinct funding streams, and independent professional networks. Although these barriers are not insurmountable, they may require diligence when conducting literature searches, tracking innovations, and identifying collaborators. It may also be the case that elements not coded for in our analysis differ across these domains such as the types of measures included in repositories (e.g., observational coding schemes for fidelity rating in behavioral health) or the kinds of non-researcher stakeholders (e.g., purveyors in behavioral health).

### Future considerations for advancing D & I science

In addition to those already noted, we offer considerations for advancing the field of D & I science based on study findings and the larger literature. First, the present study relied on the resource of a publicly available website. Websites provide essential communication about, and often access to, the resource initiative’s resources. Additionally, websites provide at least one point of access to the resource initiative for interested stakeholders. Therefore, up-to-date websites with rich content are themselves a resource to the D & I science community. Anecdotally, the reviewed websites varied widely in the extent to which they could be navigated and information easily found. Website usability issues are well-documented across a wide variety of sectors and impact the effectiveness and satisfaction with which users can engage with online materials [32–35]. More explicit attention to potential usability issues may further the goal of making websites accessible to the broadest range of D & I professionals.

The field of D & I science is rapidly expanding, and a large body of knowledge has been accumulated. Through the resources they offer, D & I science resource initiatives provide considerable opportunities to help researchers and practitioners sift through and apply this knowledge. Additionally, although promising for EBP D & I, this rapid expansion risks redundancy of efforts or potentially overlooked areas of need [10]. Our content analysis revealed that some resources are more common than others among the resource initiatives (e.g., news and updates from the field). Although we cannot say whether these resources are redundant without more information on the nature of the resource and who accesses them, we have observed a degree of resource clustering within these categories. Initiatives, particularly those with limited funding or workforce resources, may choose to put more effort into providing resources that do not already appear to saturate the field of D & I science. Our analysis also identified areas where more resources may be warranted (e.g., mentorship).

We propose that D & I science resource initiatives may also benefit from considering how their resources and characteristics align with the tenets of D & I science delineated by Glasgow and colleagues [10]. The tenets include collaboration, efficiency and speed, rigor and relevance, improved capacity, and cumulative knowledge. For instance, the interactive resources that clearly promote collaboration (e.g., workgroups, conferences) and improved capacity (e.g., mentorship) were less common than the non-interactive resources that likely promote cumulative knowledge (e.g., resource library, archived talks/slides), indicating a potential need for a shift in resource allocation. Unfortunately, without knowing the exact nature of the resources offered and stakeholder involvement in resource initiatives, it is unclear the extent to which the tenets of efficiency and rigor and relevance are well promoted. Even without such clarity, however, resource initiatives may still benefit from considering how their own resources and characteristics align with the tenets and incorporating them into strategic planning. For instance, the Society for Implementation Research Collaboration underwent a self-study with this exact purpose and determined the theme of their 2015 conference to fill a perceived gap in targeted efforts at advancing efficiency and speed [36].

### Limitations

There are several limitations to the current study in addition to those previously noted. Our content analysis was based on information gathered on publicly available websites. Websites are not static sources of information, making it possible that the results reported are not reflective of the current state of resource initiative characteristics and offerings. Rather, the data presented reflects the information available on each resource initiative’s website from October 21, 2015 to March 21, 2016. Additionally, we were not able to access all the information about some resource initiatives when membership was required to view certain parts of the website. Accordingly, this may have resulted in an underreporting of offerings for such resource initiatives. A more accurate depiction of resource initiatives, including characteristics such as non-researcher stakeholder role, and the resources they offer may require interviews with resource initiative members or leadership. However, the latter approach was beyond the scope of the modestly funded present study. Finally, although we attempted to sample from the global population of D & I science resource initiatives, it is also possible that our sampling strategies, particularly the requirement that materials be provided in English, resulted in under-sampling resource initiatives outside of the USA and Canada. In fact, the majority of resource initiatives identified were based in the USA. Future research might explicitly target non-English language websites to ensure a more comprehensive review of global resource initiatives. Additionally, given that the field uses myriad terms worldwide to refer to D & I science, we may have missed relevant search terms and unintentionally excluded resource initiatives that use those terms to refer to their efforts.

## Conclusions

The recent growth in D & I science resource initiatives suggests a rapidly expanding community focused on the adoption and sustainment of health-related EBPs. We are unaware of any previous attempt to systematically collect knowledge about these resource initiatives and examine their unique merits. Despite the limitations above, the present study reflects a considerable step forward in understanding current resources available for D & I science and the resource initiatives that promote the field. Although spanning many disciplines and health domains, D & I resource initiatives appear to reflect a single field of D & I science devoted to supporting information sharing and collaboration to improve the D & I of EBPs. Nevertheless, there exists room for improvement. Mentorship opportunities, in particular, seem to be ripe for expansion, although they may require the most intensive resources to be successful. An increase in options for virtual access to interactive resources may improve the reach of these vital resources. Additionally, as resource initiatives continue with and expand on their resources, it may be useful to consider strategic attention to the core tenets of D & I science put forth by Glasgow and colleagues [1010] to most efficiently and effectively advance the field.

## Abbreviations

D & I: Dissemination and implementation
EBP: Evidence-based practice
NIH: National institutes of health
NoE: Network of expertise
SIRC: Society for implementation research collaboration
